# A new insight into the exosome protein and lipid composition in camel colostrum and mature milk using comparative proteome and lipidomics analyses

**DOI:** 10.1016/j.fochx.2025.102729

**Published:** 2025-07-09

**Authors:** Liang Ming, Li Yi, Yisi Ai, Rimutu Ji

**Affiliations:** a College of Food Science and Engineering, Inner Mongolia Agricultural University, Hohhot 010018, Inner Mongolia, China; b Inner Mongolia Autonomous Region International Mongolian Hospital, Hohhot 010013, Inner Mongolia, China

**Keywords:** Camel milk, Exosome, Proteomics, Lipidomics

## Abstract

Camel milk exosomes from colostrum (C) and mature milk (M) were characterized using proteomics and lipidomics to elucidate their compositional and functional differences. Isolated exosomes exhibited round and cup-like concave spheres of 30 to 150 nm and higher TSG101 expression in colostrum. Proteomic analysis identified 2479 proteins, with 843 differentially expressed (357 upregulated, 486 downregulated) between C and M, enriched in PI3K-AKT signaling, endocytosis, and complement/coagulation pathways, suggesting roles in immune regulation and neonatal development. Lipidomics revealed 1783 lipid species (37 subclasses), with 149 significantly altered between C and M, including SM (d42:4; FC = 221.887) and LPE (18:3; FC = 0.139). Colostrum exosomes were enriched in sphingomyelins and glycerophospholipids, while mature milk contained higher lysophospholipids, reflecting stage-specific nutritional adaptations. These findings provide novel insights into camel milk exosome biology, highlighting their potential as biomarkers and functional food components for infant nutrition and health applications.

## Highlights

• Camel colostrum and mature milk exosomes were round and cup-like concave spheres of 30 to 150 nm.
• There were 843 and 149 differentially exosome proteins and lipid species between camel colostrum and mature milk.
• Critical lipid subclasses were triglycerides, phosphatidylcholines, and phosphatidyl ethanolamines.
• Camel milk exosomes could decrease insulin resistance through the PI3K-AKT signaling.

## 1 Introduction

Milk is a biological fluid rich in nutrients needed for the growth and development of young animals, containing protein, fat, minerals, vitamins, and various active ingredients. Due to its relatively complete nutritional structure, milk is the most complete food for digestion and absorption by newborns. The nutritional composition in milk play essential roles in growth, development, gut microbiota regulation, and disease prevention (Chen et al., 2021).

Exosomes are a type of membranous microvesicle widely present in various bodily fluids, including milk. Milk contains a high abundance of exosomes, which are produced by mammary glands, with a particle size ranging from 50 to 150 nm (Zhao et al., 2021). In 2007, milk-derived exosomes were first identified in human breast milk, and it was demonstrated that these exosomes could modulate immune responses (Admyre et al., 2007). Since then, the interest in milk exosomes has expanded to different mammals, including cows (Yang et al., 2017), buffalo, goats (Ahmed et al., 2022), camels (Shaban et al., 2023), and pigs (Ferreira et al., 2021). Milk exosomes exhibit complex compositions, containing not only proteins and lipids but also DNA and various RNA species, which may serve as potential biomarkers for certain diseases (Valadi et al., 2007). It has been confirmed that human milk exosomes are RNA-rich and can be absorbed by infants during breastfeeding (Lässer et al., 2011). Bovine milk exosomes possess a large content of miRNAs that remain stable in acidic gastric conditions, enabling intestinal absorption (Izumi et al., 2015). Reinhardt et al. (2012) determined that bovine milk exosomes included a large amount of protein, and Samuel et al. (2017) further suggested that immune-related proteins from bovine milk exosomes could mediate immune responses based on proteomic functional enrichment analysis. Yak milk-derived exosomes have been shown to enhance cellular tolerance to hypoxic conditions (Gao et al., 2019). Similarly, camel milk exosomes exhibit inhibitory effects on breast cancer cell proliferation (Badawy et al., 2018). Furthermore, porcine milk exosomes contain immune-related miRNAs that exert anti-inflammatory effects on small intestinal epithelial cells by regulating the NF-κB inflammatory signaling pathway (Xie et al., 2019).

Camel milk, comprising milk from both dromedary camels (*Camelus dromedarius*) and Bactrian camels (*Camelus bactrianus*), exhibits distinct nutritional profiles between the two species. Comparative analyses reveal significant interspecies variations in milk composition, attributable to a combination of genetic factors (breed-specific characteristics), environmental adaptations (habitat-specific influences), and management practices (rearing conditions) (Alhaj et al., 2022). Notably, camel milk possesses exceptional nutritional value and multiple benefits, establishing it as a traditional and healthy dietary staple for ethnic minority populations in northern China. Unlike other types of animal milk, camel milk offers unique advantages including high fat and protein content, combined with low cholesterol, low sugar, and abundant polyunsaturated fatty acids (Shaban et al., 2023). Research has demonstrated that camel milk lacks β-lactoglobulin and contains either no or only trace amounts of κ-casein, making it more suitable for individuals with bovine milk allergies (Cardoso et al., 2010). The fatty acid composition in camel milk is easy to digest and absorb and contains more long-chain fatty acids (C14-C18), making it healthier to drink. Furthermore, in contrast to cow milk, camel milk is rich in vitamin C, serving as the primary source of vitamin C for local herders and providing an effective alternative to fruits and vegetables for meeting vitamin C requirements (Benmeziane-Derradji, 2021). Moreover, research has shown that camel milk contains bioactive components including lactoferrin, immunoglobulin, lysozyme, and heavy chain antibodies (IgG2 and IgG3), which exhibit multiple therapeutic properties such as anti-inflammatory, immunomodulatory, and medicinal effects, particularly in alleviating diabetes, liver injury, colitis, and kidney disease (Ayoub et al., 2018; He et al., 2022; Ming et al., 2020).

Milk-derived exosomes harbor functional proteins and lipids, as evidenced by previous studies human and bovine milk exosomes (Yang et al., 2017). Nevertheless, there is limited research on exosome proteins and lipids in camel milk. This study aims to systematically analyze exosome proteins and lipids in camel colostrum and mature milk using proteome and lipidomics methods. The research results will enhance our comprehension of proteins and lipids of exosomes from camels across different lactation stages and provide a data reference for the development of camel milk products, particularly non-allergenic functional foods.

## 2 Materials and methods

### 2.1 Bactrian camel milk sample collection

Bactrian camel colostrum (0–7 days postpartum) samples were acquired from 24 healthy Bactrian camels of 4–5 years old. Parturition dates were recorded, and mature milk samples (120–125 days postpartum) were obtained from the same camels. All camels were maintained under natural grazing conditions. The Animal Administration and Ethics Committee of the Camel Protection Association of Inner Mongolia reviewed and approved the experimental protocol (Permit No. 2023-006). To minimize individual variation, milk samples from three camels were mixed, and in each group (colostrum and mature milk), eight samples were stored for subsequent experiments. This study was approved by the Inner Mongolia Agricultural University.

### 2.2 Exosome isolation from milk samples

Exosomes from camel colostrum and mature milk were extracted based on ultracentrifugation method as described by Gao et al. (Gao et al., 2019) with simple modification. Briefly, milk samples were first centrifuged at 2000*g* for 30 min at 4 °C to separate milk fat. The resulting supernatant was centrifuged at 10,000*g* for 45 min at 4 °C, followed by filtration through a 0.45 μm membrane. Subsequently, the filtrate centrifuged at 100,000*g* for 70 min at 4 °C, and crude milk exosomes were re-suspended in 1 × phosphate buffer saline (PBS, pH = 7.3) and subjected to a second ultracentrifugation (100,000*g* for 70 min). Finally, the purified exosomes were resuspended in 100 μL of pre-cooled 1× PBS for downstream analyses, including transmission electron microscopy (TEM), particle size measurement, as well as protein and lipid extraction.

### 2.3 Identification and analysis of exosomes

#### 2.3.1 TEM

Exosome samples were applied to a 400 mesh copper grid for 1 min, and the excess floating liquid was absorbed using filter paper. Samples were negatively stained with 10 μL of a 2 % uranyl acetate solution (*w*/*v*; Electron Microscopy) for 30 min, air dried, and viewed using a transmission electron microscope (Hitachi H-7500, Hitachi, Japan) at 100 kv.

#### 2.3.2 NTA

Based on NanoSight NS300 (NAT 3.1, Malvern Panalytical, UK) system to detect the concentration and size distribution of particles according to the research (Yang et al., 2017). A volume of 10 μL exosomes was diluted to 1:25–1:1000-fold in PBS to maintain a particle count between 50 and 200 per frame for real-time direct imaging and observation of specific exosome within a diameter range of 50–1000 nm.

#### 2.3.3 Western blot (WB)

Exosome proteins were mixed with the protein loading buffer, and loaded on 15 % SDS-PAGE (Sodium Dodecyl Sulfate Polyacrylamide Gel Electrophoresis). Proteins were transferred to a polyvinylidene difluoride membrane at 300 mA for 2 h (Bio-Rad Laboratories, Munich, Germany). The membranes were soaked in 5 % skim milk TBST (Tris-HCl, NaCl, Tween 20) with the protein side facing upwards and blocked for 1 h. Blots were incubated with tumor-sensitive gene 101 protein TSG101 (ABP56454, Abbkine, USA; dilution ratio was 1:1000) overnight at 4 °C and washed three times with TBST at room temperature. Then, the blots were incubated with goat anti-rabbit secondary antibody (A9169, Sigma, USA; dilution ratio was 1:5000) at room temperature for 1 h, followed by three washes. After coloring with Tianneng Western blot (WB) ultra-sensitive chemiluminescence solution (180–501, Tanon, Shanghai), imaging was performed using a VersaDoc MP4000 imaging system (Bio-Rad, Hercules, CA, USA).

### 2.4 Proteomic analysis of camel milk exosomes

#### 2.4.1 Protein extraction and digestion

Camel milk-derived exosomes were lysed using SDT buffer (4 % SDS, 100 mM DTT, 150 mM Tris-HCl pH 8.0) and were further sonicated. The lysates were centrifuged at 14,000*g* for 40 min, and the protein concentration in supernatant was determined (using the BCA Protein Assay Kit, Bio-Rad, USA). A mass of 20 μg protein from each sample was mixed with an appropriate amount of 5× loading buffer solution and then subjected to SDS-PAGE (4 %–20 % preformed gradient gel, 180 V, 45 min). Coomassie Brilliant Blue R-250 staining was performed.

Protein digestion was completed based on the method of filter-aided proteome preparation (Wisniewski et al., 2009), and were fractionated using a Thermo Scientific™ Pierce™ High pH Reversed-Phase Peptide Fractionation Kit. The C18 Cartridges (Empore™ SPE, Sigma) was used to desalt and reconstituted in 40 μL of 0.1 % (*v*/v) formic acid.

#### 2.4.2 Liquid chromatography−MS/MS analysis

LC-MS/MS analysis was performed using a Q-Exactive HF-X mass spectrometer (Thermo Scientific) with data-independent acquisition (DIA) mode. Detection mode: positive ion, primary mass spectrometry scanning range: 350–1800 *m*/*z*, mass spectrometry resolution: 120000 (@ m/z 200), AGC target: 3e6, Maximum IT: 30 ms. MS2 used to DIA data acquisition mode, with 44 DIA acquisition windows set, mass spectrometry resolution: 30000 (@ m/z 200), AGC target: 3e6, Maximum IT: auto, MS2 Activation Type: HCD, Normalized conflict energy: 30 eV. DIA data were analyzed with Spectronaut TM 14.4.200727.47784. The software parameters were set as follows: the retention time prediction type was set to dynamic iRT, interference on MS2 level correction was enabled, and cross-run normalization was enabled. All results were filtered using a Q-value cutoff of 0.01 (corresponding to FDR < 1 %).

### 2.5 Lipidomic analysis of camel milk exosome

#### 2.5.1 Lipid extraction

The lipids of camel milk-derived exosomes were isolated with methyl tert-butyl ether (MTBE) according to the procedure of Wang et al. (2022), with minor variations. Milk samples were mixed with an internal lipid standard mixture and MTBE (methanol (v/v, 5:1). After vortex mixing for 60 s, the samples were sonicated in a low-temperature water bath for 20 min and centrifuged at 10 °C for 15 min at 14,000*g*, and then collected the upper organic phase. The lipid extraction was redissolved using 200 μL 90 % isopropanol/acetonitrile solution and centrifuged at 10 °C for 15 min at 14,000*g* before mass spectrometry analysis.

#### 2.5.2 UHPLC-Q-Exactive conditions

Chromatographic analysis was using Ultra High-Performance Liquid Chromatography System (UHPLC Nexera LC-30A) with a Kinetex C18 column. Mobile phase A consisted of acetonitrile aqueous solution (acetonitrile:water = 6:4, v/v). Mobile phase B consisted of an acetonitrile-isopropanol solution (acetonitrile:isopropanol = 1:9, v/v). Injection volume wa 2 μL, column temperature was 45 °C and low rate was 300 μL/min. The elution process was as follows: 0–2 min, 30 % B; 2–25 min, 100 % B; 25–35 min. 30 % B. The injection volume was 2 μL in positive or negative mode.

The analyses were performed using electrospray ionization (ESI) in both positive and negative ion modes. After UHPLC separation, the samples were subjected to mass spectrometry analysis using a Q Exactive series mass spectrometer (Thermo Scientific™). The ESI source conditions were set as follows: Heater Temp 300 °C, Sheath Gas Flow rate 45 arb, Aux Gas Flow Rate15 arb, Sweep Gas Flow Rate 1arb, spray voltage 3.0KV, Capillary Temp 350 °C, S-Lens RF Level 50 %, MS1scan ranges: 200–1800. The mass-to-charge ratios (*m*/*z*) of lipid molecules and their fragments were acquired using the following parameters: Ten fragment spectra (MS2 scan, HCD) were collected following each full scan. MS1 has a resolution of 70,000 at *M*/*Z* 200, MS2 has a resolution of 17,500 at M/Z 200.

### 2.6 Statistics and Bioinformatic analysis

The significance of differences in exosome size and concentration was evaluated using one-way parametric analysis followed by Tukey's post hoc test using GraphPad Prism software (Inc., La Jolla, CA) with *P* < 0.05 considered as statistically significant. The graphical interface was illustrated using R software. The differentially expressed exosome proteins were screened via the Student's *t*-test and fold change (FC) values (upregulation greater than 1.5 times or downregulation less than 0.67 times). The screening criteria of significantly differentially expressed lipids were set to *P* < 0.05, variable importance in projection (VIP) > 1, and either FC > 1.5 (upregulated) or FC < 0.67 (downregulated). The statistics function ‘prcomp’ within R (https://www.r-project.org) (Chen et al., 2021) was used to complete orthogonal partial least squares discriminant analysis (OPLS-DA). The identified proteins between camel colostrum and mature milk exosomes were displayed in the Venn plot. Hierarchical clusters and volcano plots were prepared using the R package ‘ComplexHeatmap’ (v3.5.0.) and ‘ggplot2′ (v3.5.0). The functional proteins from camel colostrum and mature milk exosomes were analyzed using gene ontology (GO) annotation (https://geneontology.org/) and the Kyoto Encyclopedia of Genes and Genomes (KEGG) pathway (https://www.genome.jp/kegg/), respectively. Furtherly, the protein-protein interaction (PPI) network was analyzed using STRING (https://string-db.org/) and visualized the results in Cytocscape V3.10.3 (Shannon et al., 2003). The top 10 hub proteins were identified via CytoHubba. Additionally, the lipid-related pathways were characterized through lipid pathway enrichment analysis (LIPEA) (https://hyperlipea.org/home) to elucidate their biological relevance.

## 3 Results and discussion

### 3.1 Microstructure and WB analysis of camel colostrum and mature milk exosomes

**Fig. 1:**
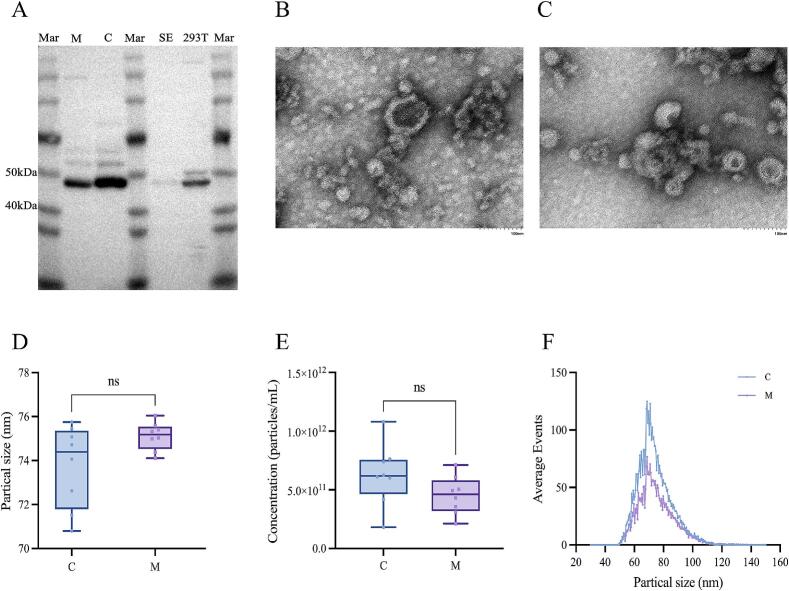
Identification of exosomal marker TSG101 in the camel colostrum (C), mature milk (M), supernatant exosomes (SE), 293T control by WB (A). TEM scanning of camel colostrum (B) and mature milk (C) exosomes. The scale bar corresponds to 100 nm. NTA analysis for size distribution (D,F) and concentration (E) in camel colostrum and mature milk.

Camel colostrum and mature milk exosomes were characterized using WB (Fig. 1A), TEM (Fig. 1B–C), and nanoparticle tracking analysis (NTA) (Fig. 1D–F). CD63, TSG101, CD8, CD81, Alix, and flotillin 1 were widely used as index proteins in WB (Lötvall et al., 2014). In this study, the exosomal marker TSG101 was confirmed in both colostrum and mature milk, and its expression was higher in the C group (colostrum) compared with the M group (mature milk) (Fig. 1A). The TEM examination revealed that the exosomes had typical round and cup-like concave spheres (Fig. 1B–C). The NTA analysis clearly showed that the exosomes extracted from camel colostrum and mature milk varied between 30 and 150 nm (Fig. 1F). The camel colostrum exosomes (73.75 ± 1.76 nm) were smaller than mature milk exosomes (75.12 ± 0.59 nm) (*P* > 0.05). This result was up to twice the size of previously reported mid-lactation milk exosomes in dromedary camels (30 nm) (Yassin et al., 2016), two times smaller than those in porcine milk (average size 152 nm) (Ferreira et al., 2021), slightly larger than human milk (50 nm) (Admyre et al., 2007). The concentration of colostrum exosomes was higher (5.02 × 10^12^ particle/mL) than mature milk (3.62 × 10^12^ particle/mL) (*P* > 0.05). Shijie (2020) reported that the concentration of exosomes in Bactrian camels was about 1.7 × 10^12^ particle/mL, which was significantly lower than our result. These differences might be because of the camel species, age, parity, environment, feeding, and lactation period.

### 3.2 Characterization of exosome proteins in camel colostrum and mature milk

**Fig. 2:**
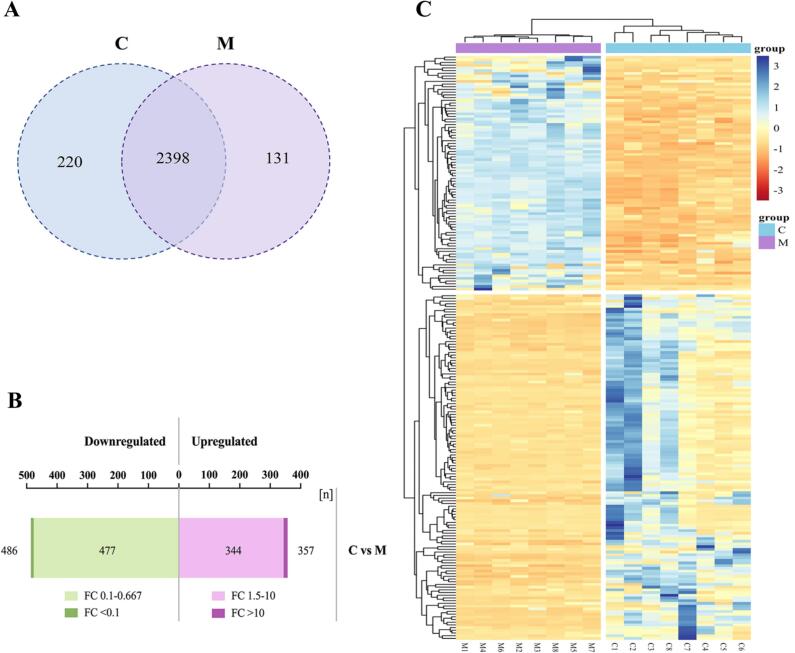
Identified proteins in camel colostrum and mature milk exosomes. Venn diagram of the exosome proteins in camel colostrum and mature milk (A). Bar chart of differentially expressed exosome proteins (B). Clustering heatmap of differentially expressed exosome proteins between camel colostrum and mature milk (C). C = camel colostrum milk; M = camel mature milk.

There 2479 kinds of milk-derived exosome proteins were identified in camel colostrum and mature milk, including 2398 shared proteins, 220 colostrum-specific proteins, and 131 mature milk-specific proteins (Fig. 2A). Exosome marker proteins including transmembrane proteins (CD9, CD63, and CD81), heat shock proteins (HSC70 and HSC90), membrane transporters (GTPases), and lipid binding proteins were detected in both camel colostrum and mature milk exosomes. Moreover, typical milk proteins, including butyrophilin and lactadherin, were present in camel milk exosomes. Butyrophilin stimulates the secretion of lipid droplets in milk, and its extracellular domain may have receptor functions. Lactadherin is a membrane glycoprotein that promotes phagocytosis of apoptotic cells. These proteins were first reported in human and bovine milk exosomes by Admyre et al. (2007) and Reinhardt et al. (2012). We also found Rab proteins in camel milk exosomes, which play an important role in vesicle fusion and trafficking events. Previous research has shown that Rab proteins can regulate cellular growth and development processes (Amir et al., 2016). These specific exosome proteins in camel milk exosomes could be used as potential biomarkers for the development of functional foods. Through our research, a large number of camel milk exosome proteins were identified, which will provide data to support further elucidating the composition, potential biological functions, and utilization of camel milk exosomes.

Furthermore, the differentially expressed exosome proteins in camel colostrum and mature milk were evaluated (Fig. 2B–C). A total of 843 differentially expressed milk exosome proteins were found (*P* < 0.05) (357 upregulated and 486 downregulated) in camel colostrum and mature milk based on the criteria of FC >1.5 (Table S1). The volcano plots of log_2_(FC) (x-axis) and their associated −log_10_(*P*-values) (y-axis) of all identified proteins in camel colostrum and mature milk were provided in Fig. S1. In our research, differences between the exosome proteomes in camel colostrum and mature milk were still greater than those found comparing the proteome of bovine mature milk and colostrum (Yang et al., 2017), human mature milk and colostrum (Yang et al., 2017), and porcine mature milk and colostrum (Ferreira et al., 2021). Additionally, some proteins exhibited more than 10 times and less than 0.1 times, respectively, in comparison with camel colostrum and mature milk exosomes (Table 1). Apoptosis, oxidative stress, inflammatory response, and immune-related proteins, such as protein glutathione peroxidase 3, heat shock 70 kDa protein 13, clusterin, IgG receptor FcRn large subunit p51 isoform X2, leucine-rich repeat-containing protein 10B were highly expressed in colostrum compared with mature milk exosomes. These proteins are significant in the healthy growth of neonates. This result is similar to previous reports in which exosome proteins derived from human, bovine, and porcine colostrum were significantly enriched in the immune response (Admyre et al., 2007; Ferreira et al., 2021; Shaban et al., 2023).

**Table 1:** Differentially expressed exosome proteins between camel colostrum (C) and mature milk (M).

Uniprot Accession	Protein name	FC value(C/M)	p-value	Regulated type
A0A8B8RDZ4	methionine aminopeptidase 1	55.943	3.040E-02	up
A0A8B6YPU0	colostrum trypsin inhibitor	29.869	3.316E-02	up
A0A8B8U7E8	epidermal growth factor receptor substrate 15 isoform X1	29.072	1.744E-03	up
A0A8B8S9C1	clusterin	27.351	5.962E-03	up
A0A8B6Y776	HLA class II histocompatibility antigen gamma chain isoform X1	19.780	2.676E-04	up
A0A8B6YEM0	heat shock 70 kDa protein 13	17.078	1.803E-05	up
A0A8B6YAG0	glutathione peroxidase 3	15.498	1.138E-02	up
A0A8B8TBK9	tetraspanin-12	14.602	4.499E-02	up
A0A8B8SK91	biglycan	14.349	4.765E-02	up
A0A8B6YFL2	stromal cell-derived factor 2 isoform X2	14.168	2.249E-02	
A0A8B7KBK0	IgG receptor FcRn large subunit p51 isoform X2	12.193	9.301E-05	up
A0A8B6YQ24	leucine-rich repeat-containing protein 10B	11.208	9.263E-03	up
A0A8B8TKG6	procollagen-lysine,2-oxoglutarate 5-dioxygenase 2 isoform X1	10.640	4.956E-02	up
A0A8B6YP51	quinone oxidoreductase PIG3	0.098	2.791E-04	down
A0A8B6YPH5	protein O-glucosyltransferase 2 isoform X2	0.095	5.615E-05	down
A0A8B8RY85	major facilitator superfamily domain-containing protein 4A isoform X1	0.088	6.286E-07	down
S9XED8	small integral membrane protein 1	0.078	5.655E-05	down
A0A8B8UIC1	LOW QUALITY PROTEIN: CMT1A duplicated region transcript 1 protein	0.077	4.124E-02	down
A0A8B8SE62	amyloid-like protein 2 isoform X1	0.060	3.789E-05	down
A0A8B8TYV5	aspartate aminotransferase, cytoplasmic	0.053	3.557E-02	down
A0A8B6YRE3	laminin subunit gamma-1	0.047	7.159E-03	down
A0A8B8TRK7	polypeptide *N*-acetylgalactosaminyltransferase 18	0.037	1.294E-04	down

### 3.3 GO analysis of the differentially expressed exosome proteins

**Fig. 3:**
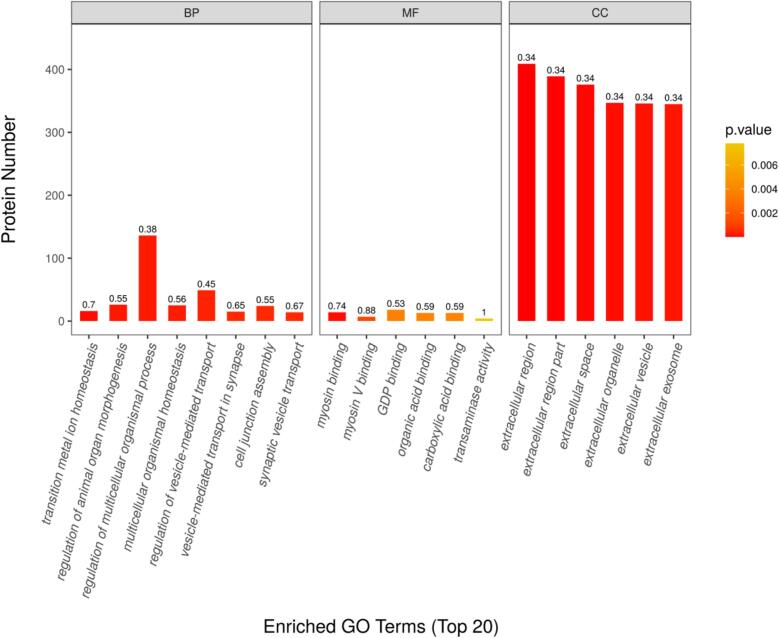
GO annotation of differentially expressed exosome proteins in camel colostrum and mature milk. BP, Biological processes; MF, Molecular functions; CC, Cellular components.

Research on exosome proteomics in both colostrum and mature milk from bovine and humans indicated that exosome proteins are mainly involved in ribosomes and regulating the actin cytoskeleton, proteins regulating cell growth, and so on (Samuel et al., 2017; Yang et al., 2017). Gene Ontology (GO) analysis was conducted to assess functional differences, with the top 20 significantly enriched terms presented in Fig. 3. The most differential GO terms among biological processes were regulation of multicellular organismal process (GO.0051241) and regulation of vesicle−mediated transport (GO.0060627). The molecular functions of these proteins were predominantly concentrated in myosin binding (GO.0017022) and GDP binding (GO.0019003). Regarding cellular components, exosome proteins mainly originated from the extracellular region (GO.0005576), extracellular space (GO.0005615), and extracellular exosome (GO.0070062), and these functional categories may be related to forming vesicular structures in the exosomes.

### 3.4 KEGG pathway analysis of the differentially expressed exosome proteins

**Fig. 4:**
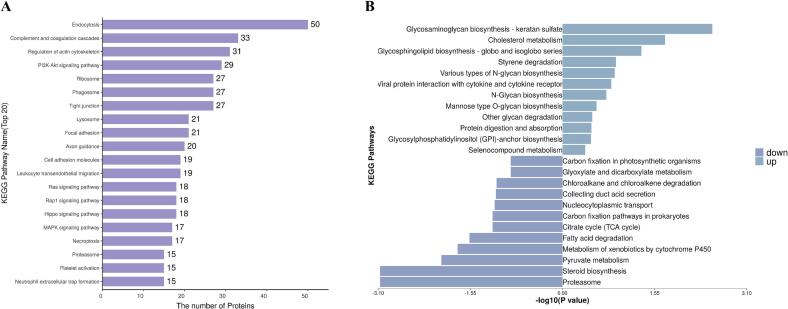
Differentially expressed proteins of KEGG enrichment between camel colostrum and mature milk exosomes. The top 20 differentially expressed proteins of KEGG (A). Pathway enrichment of differentially expressed proteins upregulated and downregulated (B).

The top 20 KEGG pathways and pathway enrichment of differentially expressed proteins are illustrated in Fig. 4A and B. Most differentially expressed milk exosome proteins were involved in endocytosis, complement, and coagulation cascades, regulation of the actin cytoskeleton, PI3K-Akt signaling pathway, and ribosomes, while some milk exosome proteins were associated with leukocyte transendothelial migration, ras signaling pathway, rap1 signaling pathway, tight junction, MAPK signaling pathway, and platelet activation. Previous studies have also confirmed that most exosome proteins from human and bovine milk are involved in these KEGG pathways (Yassin et al., 2016; Zhao et al., 2021). The endocytic pathway is “a bridge” for exchanging information between cells and the external environment and is responsible for delivering membrane proteins and phospholipids (Doherty & Mcmahon, 2009). These differentially expressed milk exosome proteins can ensure the normal progress of various cellular biological processes, such as nutrient absorption, cell migration, cell division, and growth factor receptor regulation (Scott et al., 2014).

There were 33 differentially milk exosome proteins enriched in the complement and coagulation cascades (Fig. S3), which were important components of the immune system. Previous studies reported that camel milk exosomes exhibit antibacterial and antifungal effects, modulated oxidative stress as well as may inhibit the proliferation of various cancer cell lines, including HepG2, HepaRG, CaCo2, Hl60, and PANC1 (Badawy et al., 2018; El-kattawy et al., 2021; Ibrahim et al., 2019; Shaban et al., 2023). These proteins, which participate in complement and coagulation cascades, may play a preventive and alleviating role in diseases and inflammation. The PI3K-AKT signaling pathway, which plays a pivotal role in insulin stimulation and cell survival (Huang et al., 2018), is participated in metabolic diseases such as diabetes. Some studies demonstrated the favorable effects of camel milk on diabetes through anti-inflammatory and antioxidant activities (Mirmiran et al., 2017). Our findings further confirmed that camel milk exosome proteins might reduce insulin resistance by the PI3K-AKT signaling pathway.

Furthermore, the endocytosis pathway (Fig. S2) and the complement and coagulation cascades pathway exhibited the highest number of differentially expressed proteins and the most significant enrichment. Endocytosis is a critical component of cancer hallmarks, influencing drug stability, therapeutic response, and immune cell function. Meanwhile, the complement and coagulation cascades pathway, part of the innate immune system, generates diverse effector molecules through nonspecific recognition mechanisms, playing a central role in pathogen clearance, inflammatory regulation, and maintenance of coagulation function (Zhang et al., 2020). The significant enrichment of differentially expressed proteins in these two key pathways, as identified in this study, may suggest functional differences in immune regulation, anti-inflammatory effects, and potential antitumor activity between camel colostrum-derived exosomes and mature milk-derived exosomes.

To further explore functional interactions among differentially expressed proteins, we constructed a PPI network using STRING. The network contained 740 nodes and 4004 edges, with an average node degree of 10.8 (Fig. S4). The top 10 hub proteins were evaluated using three centrality methods (Degree, EPC, and MCC) in the CytoHubba plugin, including Ribosomal protein S27A(RPS27A, albumin (ALB), ubiquitin (UBB), clathrin heavy chain 1 (CLTC), ras homolog family member A (RHOA), growth factor receptor-bound protein2 (GRB2), apolipoprotein B(APOB), catenin beta-1 (CTNNB1), and integrin beta-1 (ITGB1). These hubs are functionally linked to endocytosis, complement cascades, and PI3K-AKT signaling, consistent with KEGG enrichment results. Notably, ALB and ITGB1 were more abundant in camel colostrum-derived exsomes than in mature milk exosomes. Both proteins exhibit complementary anti-inflammatory and antioxidant properties. ALB plays regulatory roles in antioxidant and anti-inflammatory functions (Bihari et al., 2020; Tanik et al., 2020). This aligns with the observed downregulation of pro-inflammatory genes (TNFα, NFkB, TGFβ1) in HepaRG cells treated with colostrum-derived exosomes (El-kattawy et al., 2021), suggesting that ALB may synergistically suppress NF-κB signaling and oxidative stress in the tumor microenvironment. Moreover, the high abundance of lactoferrin in camel colostrum exosomes could potentiate ALB's iron-chelating and antioxidant effects, contributing to the reported anti-cancer properties of camel milk exosomes (Badawy, El-Magd and AlSadrah, 2018, Badawy, Othman and El-Magd, 2021; El-kattawy et al., 2021). ITGB1 overexpression has been shown to reduce pro-inflammatory cytokines (IL-1β, IL-6, TNF-α) and reactive oxygen species (ROS) production in LPS-injured cells (Qi et al., 2024; Song et al., 2019), suggesting its crucial involvement in tissue repair mechanisms. The remaining hub proteins collectively contribute to essential cellular processes including protein synthesis (RPS27A), lipid metabolism (APOB) (Behbodikhah et al., 2021), vesicular trafficking and endocytosis (CLTC), growth factor signal transduction (GRB2) (Wang et al., 2024), and Wnt signaling and cell adhesion (CTNNB1) (Van der Wal & Van Amerongen, 2020). This network analysis not only confirms the KEGG pathway enrichment results but also identifies key molecular players that may underlie the developmental and protective functions of camel milk exosomes.

### 3.5 Characterization of exosomes lipids in camel colostrum and mature milk

**Fig. 5:**
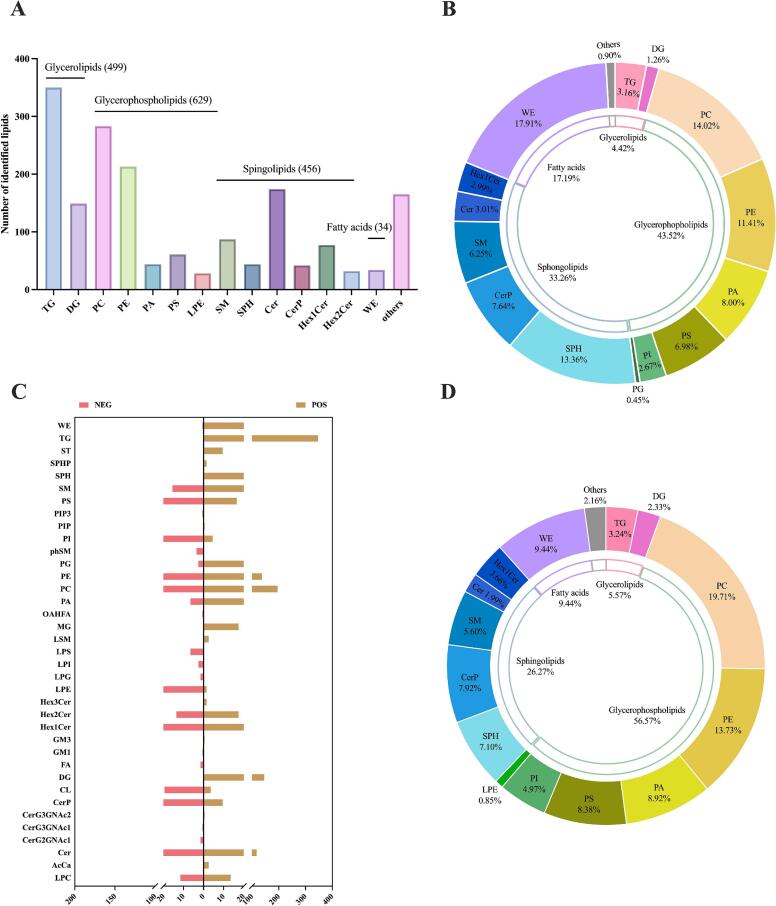
Identified lipid subclasses in camel colostrum and mature milk exosomes. Number of lipids in each subclass (A). The proportion of various lipids in camel colostrum exosomes (B). Comparison of the identified lipid numbers between POS and NEG ion modes (C). The proportion of various lipids in camel mature milk exosomes (D).

The lipidomic analysis of camel colostrum and mature milk exosomes was performed in positive (POS) and negative ion (NEG) modes. A total of 1783 lipid species were identified and classified into 37 different lipid subclasses (Fig. 5A and C), including 350 TGs, 149 DGs, 26 PCs, 28 PEs, 44 PAs, 61 PSs, 28 LPEs, 3 SMs, 44 SPHs, 174 Cers, 42 CerPs, 77 Hex1Cers, 32 Hex2Cers, and 34 WEs. The qualitative and quantitative lipid results are given in Table S2. The top five lipid classes included triglycerides (TGs,19.63 %), phosphatidylcholines (PCs, 15.87 %), phosphatidyl ethanolamines (PEs, 11.95 %), ceramides (Cers, 9.76 %), and diacylglycerols (DGs, 8.36 %) (Fig. S5).

Distinct lipid composition profiles were observed between camel colostrum and mature milk exosomes (Fig. 5B and D). In colostrum exosomes, WE, PC SPH, and PE were the predominant lipid components, while PC, PE, WE, and PA were the main lipid components in mature milk exosomes. These were completely different from the content of lipidomics in camel milk, and TG was the highest lipid in mammalian milk, such as camel, human, cow, buffalo, horse, goat, and pig (Wu et al., 2023). The level of PC in camel mature exosomes (19.71 %) was higher than colostrum milk exosomes (14.02 %), and this was higher than that of the content in camel milk (11.49 %) (Wu et al., 2023). Research reported that PC was the main source of choline (Zou et al., 2013), which is crucial for the growth and development of infants. PE was reported to be enriched in camel colostrum and mature milk exosomes, 11.41 % and 13.73 %, respectively, which is similar to the content in camel milk (Wu et al., 2023). Previous studies suggested that PE was an essential composition of mammalian cell membranes and it has been shown to enhance brain development (Zheng et al., 2023). Therefore, the high levels of PE compounds in camel milk exosomes, which were together with camel milk, may play an important role in the development of the nervous system. Interestingly, a high content of PS in camel colostrum (6.98 %) and mature milk (8.38 %) exosomes was found, and this may be related to enhanced long-term health outcomes since PS can improve nerve cell function and facilitate brain memory function (Chizhikov et al., 2020). In addition, the level of SPH was significantly higher in camel colostrum exosomes (13.36 %) compared to mature milk exosomes, suggesting this lipid enrichment may accelerate neonatal immune defense development (Nilsson et al., 2021). In contrast, the concentrations of TC, PA, and CerP showed no significant differences between the colostrum and mature milk exosome groups (Fig. 5B and D).

### 3.6 Differential analysis of lipid species in camel milk exosomes

**Fig. 6:**
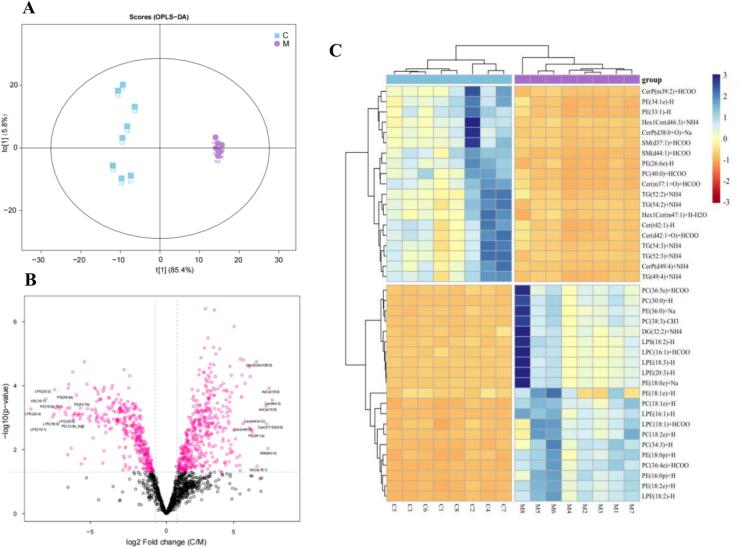
Analysis of OPLS-DA (A), Volcano plot (B), and heatmap in the camel milk exosomes (C). C = camel colostrum; M = camel mature milk.

A supervised OPLS-DA model was used to visualize the separation of lipids in camel colostrum and mature milk exosomes. Based on the OPLS-DA score plots (Fig. 6A), lipid extracts in the colostrum and mature milk groups displayed a high degree of separation. To better screen for potential lipid markers, the criteria set were OPLS-DA VIP value>1, *P* < 0.05, and either FC > 1.5 or FC < 0.67. Differential lipid analysis revealed 149 significantly different lipid species belonging to 16 lipid subclasses between the colostrum and mature milk groups (Table S3). This result suggested that most of the lipids in exosomes were the same in camel colostrum and mature milk exosomes. Furthermore, a volcano plot was constructed to exhibit the differences in lipids and to evaluate their statistical significance (Fig. 6B). This included 61 upregulated and 88 downregulated significantly expressed lipids in the colostrum compared with the mature milk group, while SM (d42:4) and LPE (18:3) showed the highest (221.887) and lowest (0.139) fold change values. This indicated that lipids with the highest multiple of differences are not the most abundant lipids, but the changes in their contents in colostrum and mature milk may have some effects on the chemical composition and nutritional value of camel milk exosome lipids (Wang et al., 2023). The LIPEA result showed that the main pathways involved in differential lipid molecules include glycerophospholipid metabolism, sphingolipid signaling pathway, and ferroptosis (Fig. S6). These pathways may drive the functional divergence between camel colostrum and mature milk exosomes.

To better visualize lipid differences between the colostrum and mature milk groups, we selected a subset of differentially abundant lipids to generate a hierarchical clustering heatmap (Fig. 6C). Camel colostrum-enriched lipid profiles revealed a predominance of glycerophospholipids (e.g., PE(34:1e)-H, PC(40:0) + HCOO), sphingomyelins (SM(d37:1) + HCOO, SM(d44:1) + HCOO), and triacylglycerols (TG(52:2) + NH4, TG(54:2) + NH4), suggesting their collective roles in enhancing membrane fluidity, supporting neonatal neuronal/immune development, and meeting the high energy demands of newborns, respectively. These findings underscore the functional adaptation of camel colostrum exosomes to early-life nutritional and physiological requirements. In contrast, camel mature milk-enriched lipids were characterized by elevated levels of lysophospholipids (e.g., LPE(18:2)-H, LPC(16:1) + HCOO), reflecting enhanced lipid metabolism efficiency, and ether-linked PCs (e.g., PC(18:1e) + H, PC(18:2e) + H), which were associated with improved oxidative stability. These findings indicate a shift toward lipid species that support long-term nutrient absorption and membrane integrity in later lactation stages.

## 4 Conclusions

This study provides the first comprehensive characterization of exosomal proteins and lipids in camel colostrum (C) and mature milk (M), revealing significant compositional and functional differences. The isolated exosomes exhibited typical morphological features, including spherical and cup-shaped structures with diameters ranging predominantly between 30 and 150 nm. Comparative analysis revealed 843 differentially expressed proteins and 1783 lipid species between colostrum and mature milk exosomes. Functional investigation of these differentially abundant molecules demonstrated their potential roles in neonatal development. These findings advance the understanding of camel milk exosome biology and offer a foundation for: developing functional dairy products by leveraging exosome-derived bioactive components; identifying lactation-stage-specific biomarkers for quality control or therapeutic applications; exploring interspecies conservation of exosome functions in milk. Future studies should validate the functional implications of the identified proteins and lipids, particularly their roles in infant nutrition and health.

## CRediT authorship contribution statement

**Liang Ming:** Funding acquisition, Writing – original draft, Conceptualization, Formal analysis. **Li Yi:** Formal analysis, Software, Writing – review & editing, Conceptualization. **Yisi Ai:** Formal analysis, Investigation, Methodology. **Rimutu Ji:** Writing – review & editing, Conceptualization, Funding acquisition, Project administration.

## Declaration of competing interest

The authors declare that they have no known competing financial interests or personal relationships that could have appeared to influence the work reported in this paper.

## Data Availability

Data will be made available on request.

## Appendix A Supplementary data

Supplementary material 1 Fig. S1 Volcano plot of the proteins in camel colostrum and mature milk exosomes. Fig. S2 Kyoto Encyclopedia of Genes and Genomes enrichment pathway map of Endocytosis pathway. Fig. S3 Kyoto Encyclopedia of Genes and Genomes enrichment pathway map of complement and coagulation cascade pathway. Fig. S4 Protein-protein interaction (PPI) network map of differentially expressed protein from camel colostrum and mature milk exosomes. Fig. S5 Percentages of numbers of lipid subclasses. Fig. S6 Pathway enrichment analysis of lipid metabolites by LIPEA. Supplementary material 1

Supplementary material 2 Table S1. Differentially expressed proteins between camel colostrum (C) and mature milk (M) exosomes. Table S2. Lipids information in camel colostrum (C) and mature milk (M) exosomes. Table S3. Differential lipids between camel colostrum (C) and mature milk (M) exosomes. Supplementary material 2
