# Supplementary material for: A new insight into the exosome protein and lipid composition in camel colostrum and mature milk using comparative proteome and lipidomics analyses
Source: Food Chem X. 2025 Jul 9;29:102729. doi: 10.1016/j.fochx.2025.102729 (PMC12369441; doi:10.1016/j.fochx.2025.102729)

Fig. S1 Volcano plot of the proteins in camel colostrum and mature milk exosomes.


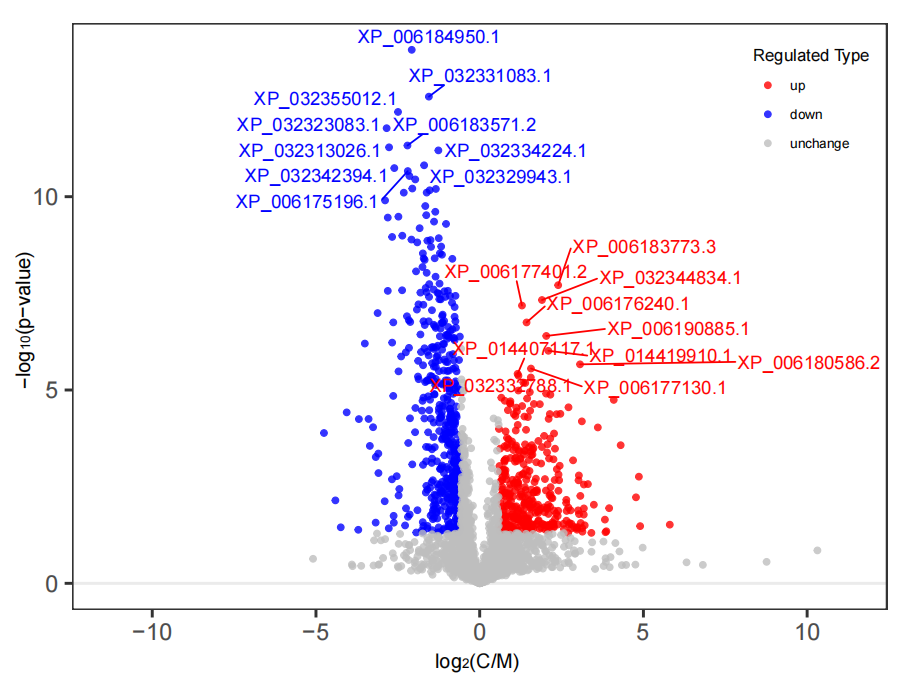


Fig.S2 Kyoto Encyclopedia of Genes and Genomes enrichment pathway map of Endocytosis pathway. The pink represents the upregulated differentially expressed proteins, blue represents the downregulated differentially expressed proteins.


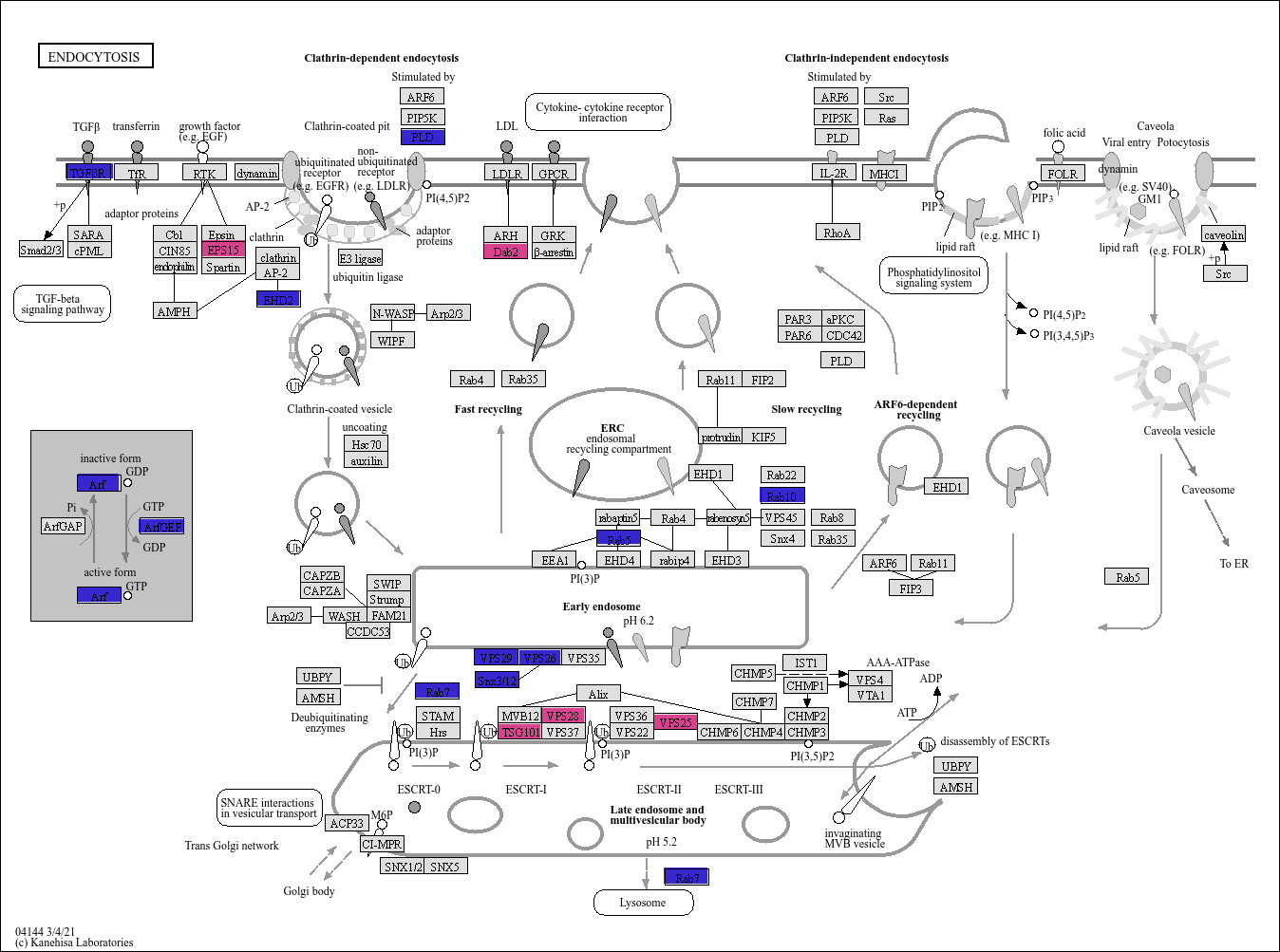


Fig.S3 Kyoto Encyclopedia of Genes and Genomes enrichment pathway map of complement and coagulation cascade pathway. The red represents the upregulated differentially expressed proteins, blue represents the downregulated differentially expressed proteins.


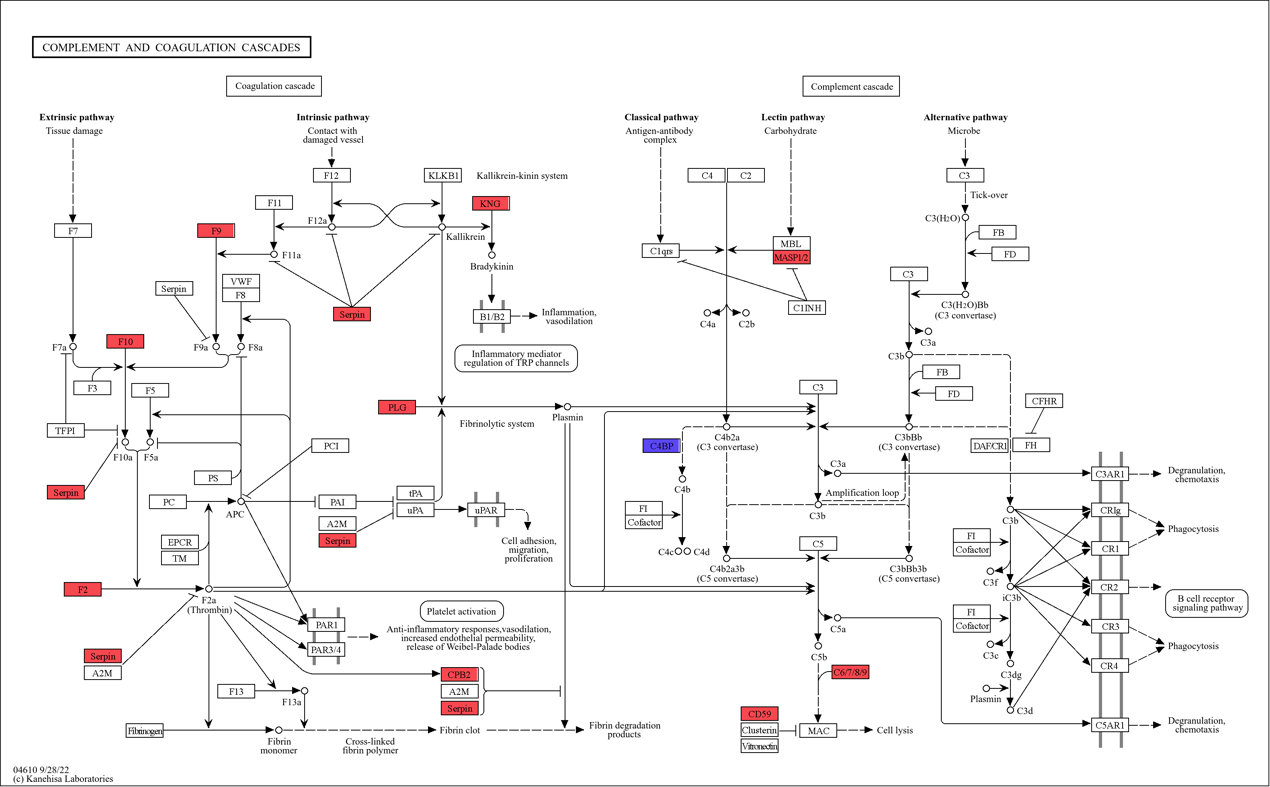


Fig. S4 Protein-protein interaction (PPI) network map of differentially expressed protein from camel colostrum and mature milk exosomes. The nod color represents the size of the degree. The node color was from green to yellow, and the corresponding degree gradually larger. The node size is proportional to its closeness centrality.


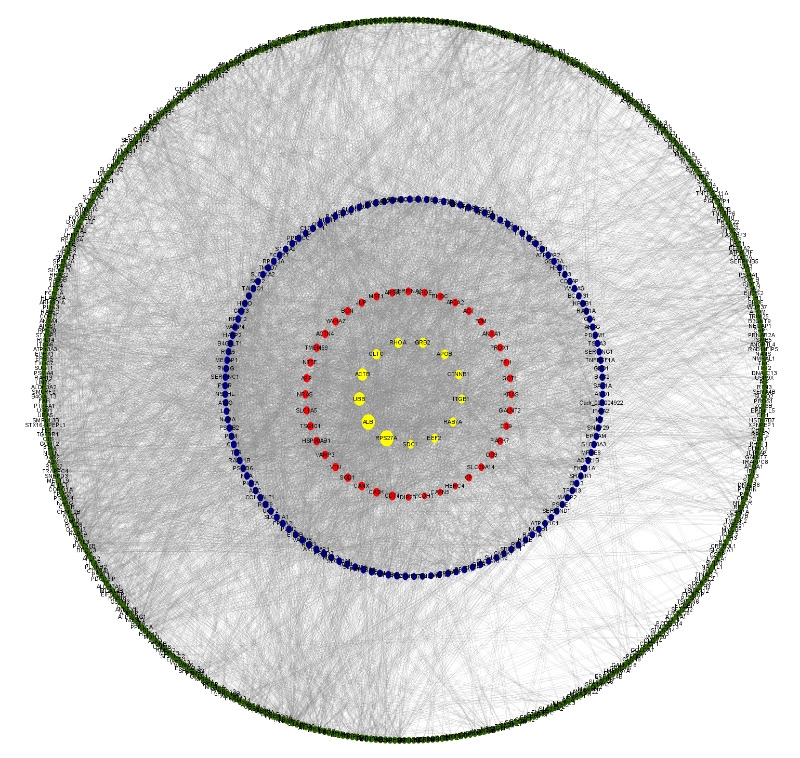


Fig. S5 Percentages of numbers of lipid subclasses.


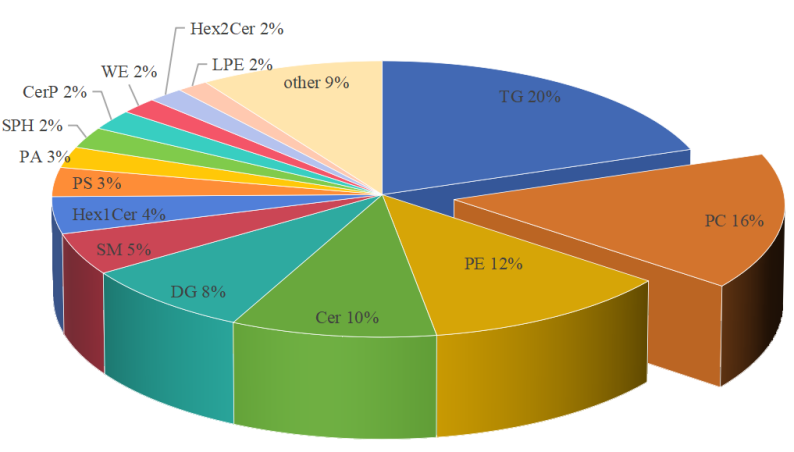


Fig. S6 Pathway enrichment analysis of lipid metabolites by LIPEA. The x-axis represents the ratio of the number of differentiated lipid molecules in the pathway to the total number of enriched differentiated lipids, converted to a percentage, and the y-axis represents the pathway name. The color of the circles indicated the significance of pathway enrichment, displayed as -Log10(*P*-value).


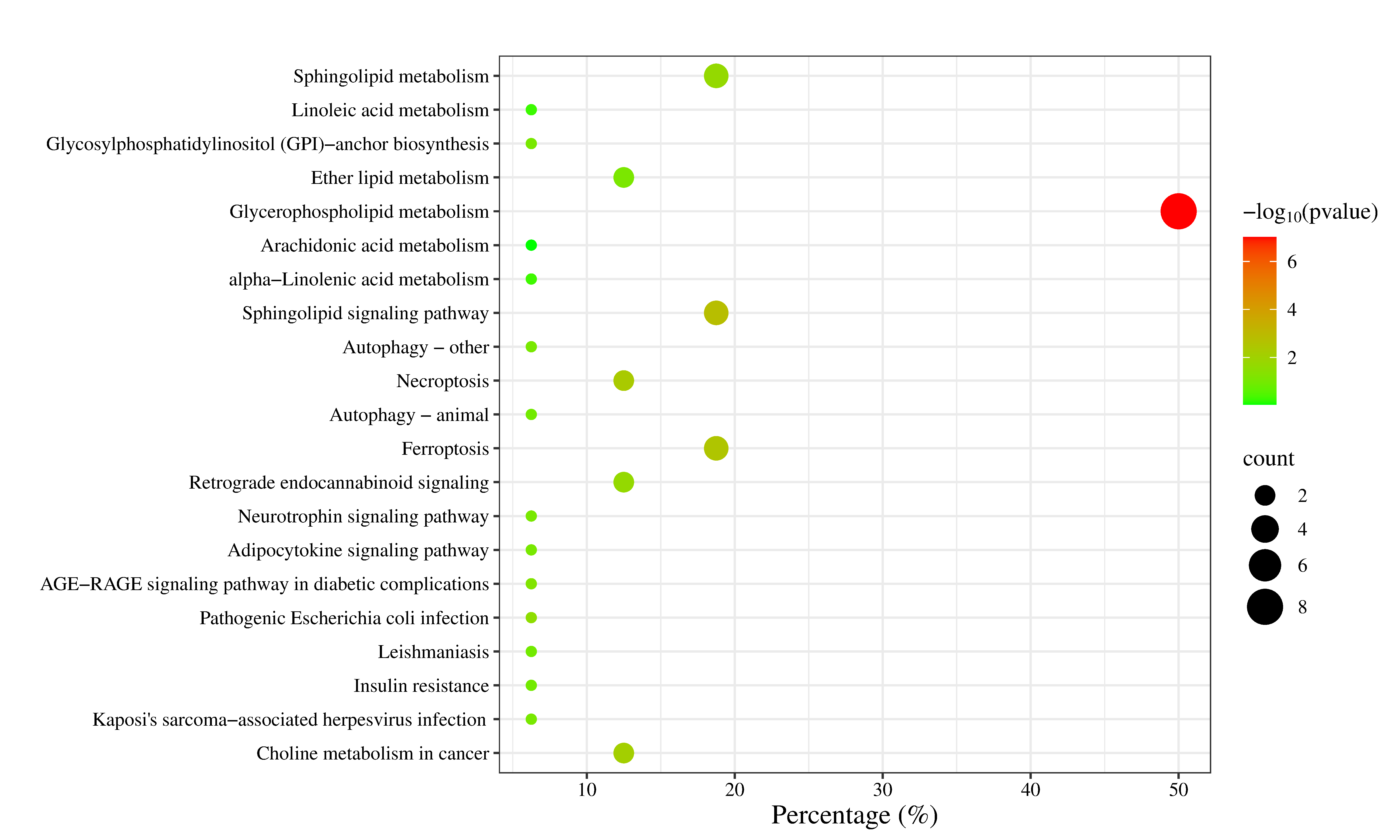

Supplement: Supplementary material 1 — Fig. S1 Volcano plot of the proteins in camel colostrum and mature milk exosomes. Fig. S2 Kyoto Encyclopedia of Genes and Genomes enrichment pathway map of Endocytosis pathway. Fig. S3 Kyoto Encyclopedia of Genes and Genomes enrichment pathway map of complement and coagulation cascade pathway. Fig. S4 Protein-protein interaction (PPI) network map of differentially expressed protein from camel colostrum and mature milk exosomes. Fig. S5 Percentages of numbers of lipid subclasses. Fig. S6 Pathway enrichment analysis of lipid metabolites by LIPEA. [file mmc1.docx]
